# The Neural Network of Orexin-A: Implications in Feeding Regulation and Obesity–Anxiety Comorbidity

**DOI:** 10.3390/brainsci16060618

**Published:** 2026-06-09

**Authors:** Jiarui Wang, Qifan Guan, Xiaokai Wei, Ying Wang, Mengyuan Li, Hongfeng Wang

**Affiliations:** 1 The College of Acupuncture and Tuina, Changchun University of Chinese Medicine, Changchun 130117, China; 24203707023@ccucm.edu.cn (J.W.); 24103707086@ccucm.edu.cn (Q.G.); 23104570126@ccucm.edu.cn (X.W.); drwangying@ccucm.edu.cn (Y.W.); 560000012@ccucm.edu.cn (M.L.); 2 Department of Physiology, China Academy of Chinese Medical Sciences, Beijing 100053, China; 3 Northeast Asia Institute of Traditional Chinese Medicine, Changchun University of Chinese Medicine, Changchun 130117, China

**Keywords:** orexin, anxiety, obesity, feeding behavior, central nervous system, corticotropin-releasing hormone (CRH)

## Abstract

**Highlights:**

**What are the main findings?**

- The orexin-A neural network centrally regulates feeding behavior and emotional responses, and its imbalance is closely linked to obesity–anxiety comorbidity.
- Key brain regions including the lateral hypothalamus, amygdala, and nucleus accumbens form the core circuit mediating the dual roles of orexin-A in metabolism and emotion.

**What are the implications of the main findings?**

- Targeting the orexin-A system represents a promising therapeutic strategy for simultaneous intervention in obesity and accompanying anxiety disorders.
- This review provides a mechanistic framework for understanding the neural basis of metabolic–emotional comorbidity and guiding future translational research.

**Abstract:**

The comorbidity of obesity and anxiety represents a complex condition with substantial health implications, exacerbating metabolic burden while compromising psychological well-being. Neurons in the lateral hypothalamus (LH) synthesize orexin-A and orexin-B, neuropeptides that orchestrate feeding behavior and energy expenditure, thereby directly regulating energy homeostasis and associated behaviors. Functioning as integrative modulators, orexins coordinate autonomic, neuroendocrine, arousal, reward, and stress circuits. Dysregulation of orexin signaling is strongly implicated in metabolic disorders, particularly obesity, as well as in psychiatric conditions including anxiety and depression, highlighting its central role in their comorbidity. This review provides a comprehensive overview of recent advances in understanding orexin-A neural circuits in feeding regulation, emphasizing mechanistic insights into the interplay between orexin signaling, energy balance, and anxiety–obesity comorbidity. Furthermore, it critically evaluates sources of heterogeneous therapeutic outcomes and outlines future strategies for precise modulation of the orexin system to restore metabolic and emotional homeostasis.

## 1. Introduction

There is a close two-way relationship between emotion regulation and metabolic homeostasis: anxiety often leads to changes in feeding behavior, and obesity is often accompanied by emotional disorders. This “anxiety–obesity” comorbidity suggests that the two share an underlying neurobiological basis. Accumulating evidence suggests that the orexin system is a key bridge connecting mood and metabolism. Animal studies have confirmed that orexin not only strongly stimulates food intake, but also widely participates in the regulation of movement, arousal and anti-anxiety responses [1]. Its dysfunction is closely related to mental diseases such as depression, anxiety and addiction [2]. At the clinical level, gene polymorphisms of orexin receptors have been found to be associated with increased susceptibility to human obesity, and serum and cerebrospinal fluid (CSF) orexin levels show a significantly lower trend in individuals with high Body Mass Index (BMI) [3,4]. These multi-dimensional physiological functions are highly consistent with the specific distribution of receptors in the paraventricular nucleus (PVN), dorsomedial nucleus (DMN) and other brain regions related to food and emotion.

However, existing research has largely examined discrete functions of orexin in isolation under physiological conditions, focusing either on metabolic regulation or stress paradigms, without establishing an integrative framework for emotional–metabolic comorbidity. More critically, the orexin system inherently possesses bidirectional regulatory properties, yet how the distinct signals mediated by OX1R and OX2R interact synergistically or antagonistically under pathological conditions remains systematically unassessed. Meanwhile, the complex interaction networks between orexin and central neuropeptides such as CRH and Neuropeptide Y (NPY), as well as pivotal metabolic hormones including insulin, leptin, and ghrelin, have been documented individually but never consolidated into a unified functional system. As a result, mechanistic explanations across different levels remain fragmented, hindering a comprehensive understanding from molecules to behavior. Methodologically, most evidence remains descriptive, limited to associations between orexin expression or activity levels, lacking rigorous causal testing; furthermore, the vast majority of studies continue to use male models, resulting in the systematic omission of sex differences as a vital biological variable.

In addition, several controversies and conflicting findings remain in the field of orexin research. For instance, patients with narcolepsy type 1 (NT1) experience excessive daytime sleepiness due to the massive loss (>90%) of orexin-synthesizing neurons. However, multiple large-cohort studies have consistently demonstrated that despite their average daily caloric intake being lower than that of healthy controls, these patients generally present with elevated BMI and increased obesity risk, which is in clear contradiction with the canonical concept of orexin’s orexigenic effect [5,6]. Furthermore, human clinical studies have yielded two conflicting reports regarding the level of orexin-A in the CSF of patients with anxiety disorders, with one reporting an increase and the other a decrease [7,8]. All the aforementioned conclusions are scientifically valid, and their inconsistency may reflect the complexity of the orexin system in orchestrating metabolic and emotional homeostasis.

In conclusion, in-depth elucidation of the underlying networks that regulate energy balance is essential for the development of novel pharmacological intervention strategies for anxiety–obesity comorbidity. Therefore, this review first summarizes the key molecular biological, neuroanatomical and pharmacological features of orexin system, and then focuses on orexin-A-mediated neural network to elaborate on the core mechanisms by which orexin peptides and their receptors regulate energy balance and emotional homeostasis. Ultimately, we will critically evaluate the current evidence linking orexin signaling to feeding behavior, aiming to highlight the great clinical potential of this system as a therapeutic target for “anxiety–obesity” comorbidity.

## 2. Overview of Orexin-A and Its Neural Circuit Connectivity

Orexin includes OXA and OXB cleaved from a common precursor, prepro-orexin. OXA consists of 33 amino acids and contains two intramolecular disulfide bonds, conferring relative stability; in contrast, OXB is a linear peptide composed of 28 amino acids. Both act through the G protein-coupled receptors OX1R and OX2R [9]. This difference in molecular conformation results in distinct affinities for the receptors. When orexin was initially discovered, it was already confirmed to exhibit excitatory activity. At the cellular level, orexin enhances synaptic transmission and neuronal activity, upregulates intracellular calcium levels, and promotes synaptic potentiation of multiple neuronal types in the brain. Two G protein-coupled receptors mediate the cellular effects of orexin, and upon activation, these receptors can couple to multiple downstream signaling pathways including Gs, Gq, and Gi [10]. Moreover, at the synaptic level, single-cell transcriptomics studies have confirmed that the ventral striatum–ventral pallidum complex (VS-VP Complex) in the telencephalon inhibits orexin neuron excitability through GABAergic input [11]. This indicates that orexin is subject to bidirectional circuit regulation at both cellular and synaptic levels.

Orexin is primarily produced in the preoptic area (PFA), dorsomedial hypothalamic nucleus (DMH), and LH regions of the hypothalamus and its axonal projections are extremely extensive and densely target the ARC, PVN and other core centers that regulate feeding behavior and energy homeostasis, thereby systematically integrating functional activities of downstream brain areas. Orexin receptor expression has obvious regional specificity: OX1R is preferentially distributed in areas related to emotion, stress and reward, including the ventral tegmental area (VTA), amygdala (AMY), and bed nucleus of the stria terminalis (BNST) [12,13]. However, OX2R mainly mediates arousal within the tuberomammillary nucleus (TMN) and basal forebrain (BF) [14,15]. This difference in affinity and spatial distribution lays the foundation for the functional diversity of orexins.

**Figure 1:**
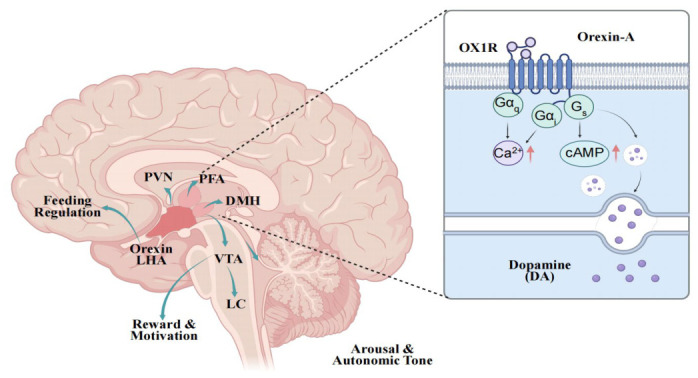
Schematic diagram of the neuroanatomical distribution of the Orexin system and its major projection pathways. Orexin neurons were mainly distributed in the lateral hypothalamic area (LHA), the perifornix area (PFA) and the dorsolateral hypothalamic nucleus (DMH). The figure shows the key projections of Orexin neurons to the whole brain: (1) to the paraventricular nucleus (PVN) to regulate Feeding behavior; (2) projection to the ventral tegmental area (VTA) to regulate Reward & Motivation; (3) projections to the locus coeruleus (LC) and brainstem areas regulate Arousal & Autonomic Tone. The upper right inset shows the mechanism of binding of Orexin-A and Orexin-B neuropeptides to their G protein-coupled receptors, OX1R and OX2R, at the postsynaptic membrane.

In summary, the unique organization of glutamatergic and GABAergic synapses on Orx neurons, together with the intricate interaction network between orexin and adjacent neuronal populations, provides a functional substrate for the potential roles of orexin in the regulation of key physiological functions in both animals and humans (Figure 1).

## 3. Central Integrative Mechanisms of Orexin: Dual Regulation of Metabolism and Emotion

### 3.1. Orexin Integrates Central Afferent Signals to Regulate Motivated Behavior

Low glucose is the most primitive, immediately available energy signal. Yamanaka used electrophysiological recordings from isolated hypothalamic brain slices to discover that extracellular glucose bidirectionally regulates orexin neuron excitability: high glucose induces hyperpolarization and suppresses firing, while low glucose triggers depolarization and increases neuronal firing frequency [16]. Paradoxically, insulin—the primary in vivo glucose-lowering hormone—does not directly induce these electrophysiological changes [17]. However, functional experiments in diabetic pathological models revealed that insulin can dose-dependently inhibit spontaneous firing of these neurons, an effect that is completely abolished by pre-perfusion with the PI3K inhibitor wortmannin or the Akt inhibitor MK-2206, indicating that insulin’s inhibitory effect on orexin neurons requires indirect regulation via the PI3K-Akt pathway [18].

In addition, leptin and ghrelin are also involved in the regulation of orexin neurons. Increased adiposity leads to elevated circulating leptin levels. Early studies reported that leptin, after crossing the blood–brain barrier, could hyperpolarize orexin neurons [19]. However, recent studies have confirmed that leptin receptor (LepR) mRNA is barely expressed in orexin neurons. The inhibitory effect of leptin is therefore not mediated through a direct action on orexin neurons, but rather through LepRb-positive neurotensin (Nts) interneurons located in the lateral hypothalamic area, which indirectly suppress orexin neuronal activity [20]. It should be clarified that under normal body fat levels, leptin still indirectly inhibits orexin neurons and downregulates their baseline excitability. When low leptin signaling is transmitted, this inhibition is relieved, and the excitability of orexin neurons increases.

Ghrelin is a key gut–brain signaling molecule secreted by the oxyntic glands in the gastric fundus during fasting. Early studies have confirmed that the orexigenic effect of ghrelin depends on an intact orexinergic signaling pathway [21]. After ghrelin’s entry into the central nervous system, ghrelin targets growth hormone secretagogue receptor (GHSR) expressed on NPY neurons within the arcuate nucleus and on orexin neurons in the lateral hypothalamic area (LHA). Electrophysiological recordings have shown that ghrelin–GHSR binding induces membrane depolarization and increases the firing frequency of orexin neurons [22]. Optogenetic experiments further confirm that ghrelin activates the dopaminergic pathway in the VTA of the midbrain via orexin neurons, which converts hunger signals into motivational approach toward high-reward foods [23], revealing the direct association between gut–brain signals and motivated behavior.

Notably, these three signaling systems do not operate independently but instead form a multi-layered interaction on orexin neurons to achieve integrated regulation. Mavanji et al.’s review systematically elaborates on this interaction: glucose sensing determines the immediate firing capacity, leptin sets the baseline range of excitability, and ghrelin provides phasic signals that trigger the initiation of feeding behavior.

### 3.2. Centrally Evoked Sympathetic Nervous Responses Mediated by Orexin

Pharmacological studies have shown that Orexin-A plays a complex dual role in regulating body energy balance through the sympathetic nervous system (SNS). Centrally, Orexin-A strongly promotes transient feeding behavior through multiple hypothalamic nuclei (especially OX1R signaling within the MPOA) mainly during the resting period (light period) [15,24]. However, this orexigenic effect did not lead to weight gain. Conversely, in the obese state, this system also exhibits adaptive remodeling of receptor hyperresponsiveness [25].

This seemingly paradoxical phenomenon reveals the core feature of the Orexin system: promoting energy expenditure is its dominant physiological function [26,27]. Mice deficient in Orexin signaling become obese due to a significant decrease in spontaneous physical activity (SPA) and thermogenesis from non-exercise activity (NEAT) despite reduced food intake [28,29]. Central Orexin-A can rapidly activate a wide network of brain regions, strongly drive SPA and NEAT, and its effect on promoting energy expenditure even overwhelms its effect on promoting food intake [30,31].

In addition, Orexin-A also drives metabolic thermogenesis by directly maintaining sympathetic tone. It can not only increase sympathetic discharge and thermogenesis of brown adipose tissue (BAT), but also induce “Browning” of white adipose tissue (WAT) under the regulation of VMH-BMP8B upstream signal [32,33]. Interestingly, this strong thermogenic activation constitutes A negative feedback regulatory loop for feeding—orexin-A-mediated increase in body temperature raises the temperature set point, thereby temporarily inhibiting subsequent feeding behavior before thermal equilibrium is reached [34,35]. At the same time, it can also enhance glucose uptake by skeletal muscle [36].

**Figure 2:**
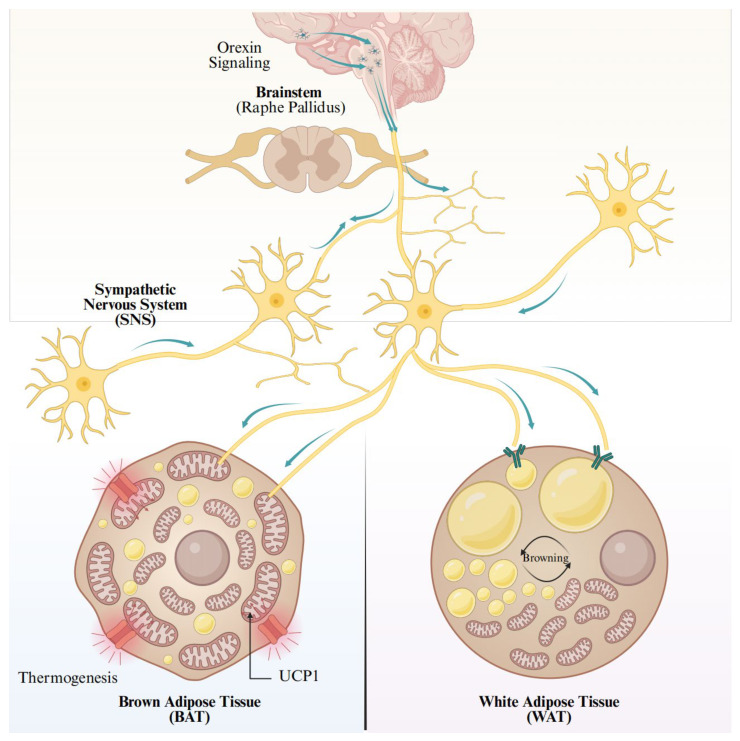
Orexin regulates thermogenesis and Browning of adipose tissue via the sympathetic nervous system. This figure depicts the multilevel signaling pathways that descend from Brainstem/Raphe Pallidus to the spinal cord to activate the sympathetic nervous system (SNS). Orexin signaling activates the upstream pathway, leading to metabolic changes in peripheral adipose tissue: (1) the up-regulation of mitochondrial uncoupling protein 1 (UCP1) in brown adipose tissue (BAT, left), which promotes Thermogenesis; (2) “Browning” of white adipose tissue (WAT, right), which is characterized by changes in lipid droplets and increased mitochondria, increases energy expenditure.

In summary, despite its ability to stimulate short-term feeding, Orexin-A’s property to consistently promote energy expenditure by “driving physical activity” and “sympathetic thermogenesis” establishes it as a central hub for metabolic homeostasis, making it a highly potential therapeutic target for the intervention of obesity (Figure 2).

### 3.3. Peripheral Effects Mediated by Orexin–Neurotransmitter Interactions

The regulation of stress-induced eating is primarily controlled by the hypothalamic–pituitary–adrenal (HPA) axis and modulated by multiple neuropeptides. Mounting evidence from the last decade has shown that orexins engage in complex interactions with neuropeptides including CRH and NPY, forming a cross-level synergistic CRH-orexin-NPY network that is involved in the regulation of emotion and metabolism [37,38].

Stress exposure acts as a key trigger for the development of anxiety-like behaviors and abnormal feeding behaviors. The report that the orexin system mediates stress-induced anxious binge eating provides the earliest evidence for the involvement of orexin in mediating anxiety–obesity comorbidities [39]. Studies have shown that CRH can directly depolarize and activate orexin neurons through CRHR1 [40]. Conversely, orexin can also significantly up-regulate CRH expression in the PVN [41,42]. This functional interaction has a significant stress phase dependence. In the early stage of acute stress, the rapid release of CRH strongly inhibits orexin activity through CRHR1, leading to stress anorexia [43,44]. However, under chronic stress, persistently high CRH levels induce orexin neurons to adaptively up-regulate OX2R expression, thereby overcoming the inhibitory effect [45,46]. This shift activates mesolimbic pathways that promote reward-driven feeding of calorie-dense foods.

NPY and orexin constitute a functionally coupled downstream effector network. Functionally, exogenous NPY perfusion markedly reduced the firing frequency and elicits membrane hyperpolarization of LH neurons, confirming direct inhibitory effects of NPY. This inhibition was almost completely abolished upon simultaneous blockade of Y1 and Y5 receptors, indicating that NPY mediates its suppression of orexin neurons via both Y1 and Y5 receptors [47,48]. Conversely, whole-cell patch-clamp recordings in acute brain slices show that exogenous orexin-A perfusion significantly elevates the firing frequency and membrane depolarization of NPY/AgRP neurons [49]. This effect is partially blocked by OX1R antagonists, demonstrating that orexin neurons exert positive regulation on NPY/AgRP neurons. Thus, the functional imbalance of the bidirectional circuit and resultant homeostatic disruption may act as the core neural substrate driving stress-induced eating and anxiety-like behaviors.

## 4. Basal Metabolic Phenotypes and Cardiometabolic Traits in Orexin-Deficient Humans and Mice

Clinical studies have shown that high levels of Orexin-A in the morning are positively correlated with good lipid and insulin profiles [50]. Conversely, reduced Orexin signaling, such as downregulation of OX1R expression in adipose tissue in obesity, leads to reduced metabolic efficiency [51]. Experiments have shown that orexin-deficient mice and narcolepsy patients show significant metabolic abnormalities, including reduced basal metabolic rate (BMR), impaired insulin secretion, and increased waist-to-hip ratio (WHR), suggesting that orexin deficiency may preferentially disrupt energy metabolism by remodeling adipose distribution and body composition, rather than regulating obesity merely through feeding control [52,53].

In addition, Orexin plays a key role in maintaining the circadian coordination of autonomic nervous and cardiovascular systems, as well as emotion regulation. Orexin deficiency not only weakens the body’s autonomic response to emotional stimuli, but also leads to the circadian rhythm disorder of the cardiovascular system, which is typically manifested as a “non-dipper” nocturnal blood pressure pattern (nocturnal blood pressure reduction is absent) [54]. This abnormality is accompanied by a decrease in baroreflex sensitivity (BRS) and heart rate variability (HRV), consistent with the features of autonomic impairment associated with anxiety [55].

Cross-species analyses demonstrate that orexin deficiency elicits complex and dynamic regulatory effects on metabolic homeostasis, cardiovascular rhythmicity, and autonomic reactivity. Orexin-deficient mice generally exhibit a highly consistent metabolic phenotype, characterized by reduced BMR and remodeled fat distribution [56]. In contrast, findings concerning cardiovascular parameters in these mice remain inconsistent. Some studies demonstrate a significant reduction in basal blood pressure during wakefulness, whereas others detect no appreciable difference compared with wild-type mice [57,58]. Additionally, heart rate during both awake and sleep periods exhibits heterogeneity [59,60,61]. Conversely, narcoleptic patients display marked heterogeneity in metabolic phenotypes: some studies indicate increased BMI and body fat ratio, but their BMR may remain normal, slightly decreased, or unchanged; other studies fail to detect significant fat distribution remodeling, with only lean mass reduction rather than simple obesity. Furthermore, patients maintain normal basal blood pressure during the awake period, with only a slight elevation during sleep, and their heart rate changes, similar to those in mice, show no consistent pattern [62,63].

Differences between rodents and humans in circuit specificity, neuroanatomy, and behavior may well account for the above discrepancies. In fact, the total number of orexin neurons is estimated to be approximately 3000 in the rat brain and 70,000 in the human brain [64]. Immunohistochemical studies reveal that the density distribution of orexin neurons differs between rodents and humans. Rodents show far lower projection density of orexin neurons toward the prefrontal cortex compared with humans [65]. Moreover, behavioral differences also play a key role. Rodents are nocturnal, with feeding and arousal concentrated during the dark phase, when hypoglycemia readily activates orexin neurons. In humans, by contrast, orexin activity fluctuates mildly, and postprandial blood glucose variations exert a more prominent influence on orexin function [66]. Consequently, orexin-deficient rodents may be more susceptible to obesity and exhibit a relatively consistent metabolic phenotype, while in humans, such deficits often manifest as heterogeneous metabolic phenotypes accompanied by comorbidities involving metabolism and mood—likely due to the greater complexity of human neural networks, behavioral patterns, and environmental influences.

Meanwhile, regardless of species, orexin deficiency consistently leads to attenuated circadian rhythm fluctuations, impaired autonomic nervous responses to emotional stimuli, and anxiety phenotypes in some human patients [67]. Collectively, these findings highlight that the orexin system fulfills a core function in integrating metabolic, emotional, and circadian regulatory networks, rather than merely sustaining sympathetic tone.

## 5. Effects of Orexin on Obesity–Anxiety Comorbidity

### 5.1. Causal Associations of Orexin in Anxiety–Obesity Comorbidity

Obesity and anxiety are highly comorbid, with a significantly higher prevalence of anxiety among obese individuals than non-obese individuals; excess body weight and elevated anxiety frequently co-occur. In patients with severe mental disorders, over 32% exhibit eating disorders and approximately 16.1% have comorbid obesity [68]. However, the exact mechanisms underlying the relationship between obesity and anxiety are still not clear and the determination of such mechanisms could be very helpful in developing pharmacological therapy to address both conditions. Neuroimaging has demonstrated that both high-fat diet and chronic stress concurrently modify neural activity in the hypothalamus and limbic system, as well as microglial activation (neuroinflammation) in the hippocampus [69]. These findings suggest a strong bidirectional facilitatory relationship between obesity and anxiety: obesity triggers anxiety through systemic inflammation and stress, whereas anxiety drives emotional eating and aggravates obesity, forming a reciprocal cycle bridged by feeding behavior. Specifically, high-fat feeding significantly upregulates prepro-orexin mRNA and orexin-A levels, while repeated stress leads to substantial release of orexin-A and enhances the activity of orexin neurons [70,71]. These findings indicate that high-fat diet and chronic stress, as major environmental triggers for comorbidities, converge on the overactivation of the orexin system, suggesting that the orexin system serves as a common causal hub linking metabolic dysregulation and mood disorders.

Mechanistically, hyperactive orexin signaling drives both metabolic disturbances and emotional disorders through OX1R. On one hand, orexin-A enhances stress-induced activation of the HPA axis and sympathetic nervous system via a CRH-dependent pathway mediated by OX1R. Pharmacological studies have shown that microinjection of orexin-A into the lateral ventricle or PVN of rats elevates plasma Adrenocorticotropic hormone (ACTH) and corticosterone levels in a dose-dependent fashion. Prior administration of the selective OX1R antagonist SB-674042 completely abolishes this effect [72]. These functional blockade experiments confirm that endogenous orexin is a necessary mediator for stress-evoked activation of the HPA-sympathetic axis. On the other hand, orexin-A signaling via OX1R along the projection pathway toward the PVN potentiates motivational seeking for highly rewarding food. Treatment with OX1R antagonists GSK1059865 reduces binge-like consumption of high-fat diets without affecting normal feeding behavior [73]. These findings highlight the critical role of OX1R in emotional eating. In summary, dysregulation of the orexin system is not merely a comorbid phenomenon but rather a causal hub linking neuroendocrine drive to the obesity–anxiety phenotype.

### 5.2. Orexin Signaling in Obesity and Circadian Metabolic Regulation

Obesity is essentially the result of a long-term imbalance between energy intake and expenditure. Unlike other single-function brain–gut peptides, Orexin uniquely exerts positive regulatory effects on both food intake and energy expenditure (by maintaining arousal) [74]. A high-fat diet disrupts this normal temporal coupling of “activity-feeding” by selectively inhibiting the spontaneous activity of animals during the active phase [75].

Upon close scrutiny of this coupling, interaction reveals that the regulatory effect of orexin exhibits metabolic-state dependence: under fasting conditions, the VS-VP Complex attenuates its inhibitory tone in response to food-proximate cues, thereby boosting orexin neuron excitability to peak firing activity. This cascade enhances arousal and appetitive drive toward food, with the intensity of food-seeking motivation positively correlating with the degree of hunger [76]. Conventional consensus holds that orexin neuronal excitability declines gradually throughout feeding and falls back to baseline upon satiety. However, recent research revealed that orexin neuronal activity rapidly returns to baseline before food ingestion initiates [77]. Collectively, these observations suggest that the core function of orexin is to promote food-directed motivational behaviors instead of directly eliciting consummatory feeding.

Notably, the orexin-driven modulation of feeding motivation is not uniformly distributed over time, but rather is tightly regulated by the circadian rhythm. Circadian clock genes govern adipogenesis, lipolysis, and thermogenesis, positioning orexin as a critical mediator linking behavioral states to metabolic rhythms. Orexin-A secretion is highly circadian (peaking during arousal/activity), and the expression of OX1R and OX2R in the hypothalamus is synchronized with circadian clock genes [78]. Anatomically, Orexin neurons have bidirectional projections to the suprachiasmatic nucleus (SCN), the master circadian clock, and are directly involved in phase resetting and circadian rhythm-dependent regulation. Once Orexin signaling is impaired (as in narcolepsy or gene knockout models), this rhythmic coupling breaks down [79,80].

In addition, sleep or rhythm disturbance can trigger significant abnormal glucose and lipid metabolism. Orexin regulates systemic metabolism not only through rhythmic output from the central SCN, but also through peripheral mechanisms, such as rhythmically expressed OX2R in the liver and the synergy between clock genes and nuclear receptors such as PPARα in the transcriptional control of lipid metabolism [81]. Taken together, the Orexin system provides a solid mechanism for “chronotherapy” in obesity intervention by precisely coordinating central rhythms with peripheral metabolic homeostasis.

### 5.3. Orexin Signaling in Anxiety and Reward Circuitry

Numerous animal and human studies have shown that orexin signaling is involved in anxiety and fear-related neural circuits that overlap with the reward pathway. Genetic studies have found that non-synonymous variants in the orexin receptor gene, specifically OX1R, are risk factors for panic disorder and agoraphobia. Increased orexin-A levels in CSF and serum have been reported in patients with panic disorder and anxiety [8].

Orexin projections from LH directly regulate mesolimbic dopamine circuits [82]. Activation of VTA dopamine neurons can enhance synaptic plasticity and increase the release of dopamine in the nucleus accumbens (NAcc), thus enhancing reward-seeking behavior [83,84]. Alcohol exposure strongly activates this pathway [85]. In contrast, dynorphin κ-opioid receptor signaling inhibits dopamine neuronal activity, providing a counterregulatory balance [86].

Projection-specific effects further shaped anxiety responses. Orexin preferentially enhances dopamine neurons projecting to the VTA, whereas dynorphin more effectively inhibits projections to the basolateral amygdala (BLA). Social stress activates glutamatergic neurons in the VTA and strengthens VTA-BLA connectivity, leading to anxieties [87,88]. Upregulation of OX1R expression within the BLA circuit during stress amplifies the anxiety response after orexin-A exposure.

**Figure 3:**
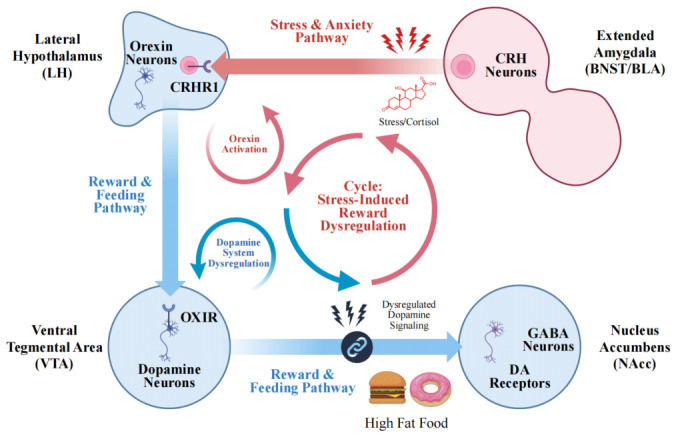
Interaction Model of Key neural circuits mediating the comorbidity of Obesity and Anxiety: The Vicious Cycle of stress and reward System. This figure demonstrates the interaction between the lateral hypothalamic area (LH), ventral tegmental area (VTA), nucleus accumbens (NAcc), and BNST/Amygdala. The red pathway indicates the stress/anxiety pathway: stress stimuli, such as elevated Cortisol, activate CRH neurons in the BNST/Amygdala, which in turn enhance the activity of Orexin neurons in the LH region via CRHR1 receptors. The blue pathway represents the reward/feeding pathway: activated Orexin neurons project to the VTA, resulting in Dopamine dysregulation, which in turn affects the NAcc and promotes high-fat food craving and compulsive eating. This “stress–feeding” cycle sheds light on the neurobiological mechanisms underlying susceptibility to obesity in anxious states.

Within the BNST, orexin-A can increase CRH activity and induce anxiety-like behavior in the elevated plus-maze paradigm [89]. OX1R signaling enhances the excitability of CRH neurons and promotes compulsive reward-seeking behavior, especially during alcohol withdrawal. Knockdown of OX1R reduces alcohol intake and anxiety behavior, whereas OX2R exerts a complementary regulatory role [90,91] (Figure 3).

The translational relevance is further supported by a human experimental model using CO_2_ inhalation. Selective OX1R antagonists such as JNJ-61393215 reduce panic symptoms without major cardiovascular side effects, whereas alprazolam additionally suppresses autonomic responses [92]. Another antagonist, ACT-539313, showed a slight anti-anxiety trend, but with limited statistical significance [93]. Differences in pharmacokinetics may explain these results: JNJ-61393215 preferentially targets the BNST/BLA circuit, whereas ACT-539313 more strongly affects the VTA-NAcc reward pathway [94]. Direct comparisons suggest that selective OX2R antagonists alone have little anti-anxiety effect [95], which further cements OX1R as the primary therapeutic target for anxiety regulation.

### 5.4. Orexin Mediates Obesity–Anxiety Comorbidity in a Sex-Dimorphic Manner

Sex-dependent factors contribute to the complex pathogenesis of obesity–anxiety comorbidity. For instance, although obesity is more frequently diagnosed in females than males, males tend to develop type 2 diabetes mellitus at a younger age and a lower BMI. The prevalence of stress-related psychiatric disorders in females is twice that in males [96]. Nevertheless, neonatal maternal deprivation exerts more prominent impacts on male animals than on females [97]. This complexity is consistent with the widespread sex dimorphism observed in the central pathways governing energy homeostasis in animal studies. Collectively, these findings indicate that the inherent sex dimorphism of the orexin system is a critical factor mediating anxiety–obesity comorbidity.

Previous studies have demonstrated that the promoter region of the orexin gene contains putative estrogen and androgen response elements [98]. Moreover, in female rodents, orexin mRNA levels exhibit dynamic fluctuations across the estrous cycle, reaching a peak during proestrus when estrogen levels are at their maximum. Ovariectomy leads to reduced orexin expression, whereas 17β-estradiol (E2) replacement therapy restores its expression level [99]. In males, testosterone mostly acts indirectly on the orexin system via endogenous estrogen produced through aromatization of testosterone. Such disparities in upstream hormonal regulation consolidate the sex dimorphism of orexin at the input level. In addition, sex differences are also evident in orexin receptor expression. Studies indicate that female rats exhibit higher baseline OX1R expression in key stress-related nuclei, including BNST and LC; the baseline expression level of OX1R may be higher than that in male rats [100]. More importantly, these receptors possess greater post-receptor coupling efficiency. When exposed to stimuli of the same intensity, female orexin neurons may exhibit stronger intrinsic excitability or more sustained transcriptional responses, which further amplifies sex differences in downstream signaling.

At the functional level, under the continuous action of estrogen, female orexin neurons exhibit higher activity and more abundant central OX1R expression. The overactivated signaling is simultaneously transmitted to multiple parallel pathways: one pathway projects via the BNST/LC to the anxiety-related corticolimbic circuit, continuously inducing anxiety and hypervigilance; another pathway drives the sympathetic nervous system via the PVN, which may induce stronger or more persistent cardiovascular responses in females. Meanwhile, orexin projects to the reward circuit of the VTA-nucleus accumbens, generating uncontrollable craving for high-energy palatable foods. The synergistic effect of the three pathways mimics the stress-induced emotional eating phenotype [101]. Over time, the abdominal obesity resulting from this process leads to leptin resistance and low-grade systemic inflammation, and these peripheral signals in turn cross the blood–brain barrier, continuously disrupting the normal regulation of orexin neurons and further promoting the progression of comorbidities [102].

## 6. Clinical Translation and Related Challenges of the Orexin System

The bidirectional deteriorative mechanisms between obesity and anxiety have been systematically elucidated. The comorbidity of these two conditions severely impairs patients’ quality of life and social functioning. Although conventional treatments can partially alleviate symptoms, they commonly suffer from limited efficacy and prominent adverse effects. First-line anxiolytic agents including SSRIs and benzodiazepines frequently induce body weight gain and increase the risk of metabolic syndrome [103]. Several anti-obesity pharmaceuticals have been withdrawn from the market owing to psychiatric adverse events such as anxiety and depression, with rimonabant (a cannabinoid CB1 receptor antagonist) serving as a representative example [104]. Such therapeutic contradictions suggest that treating obesity and anxiety requires targeting shared neural circuits, and the orexin system fulfills this therapeutic potential. Hypothalamic orexin neurons project extensively throughout the central nervous system and act as master regulators of feeding, emotion, and motivation. Advances in experimental techniques enable precise manipulation of orexin neurons and their downstream target brain regions, rendering this system an optimal anatomical and functional hub linking metabolic and affective regulation [55].

However, the complexity of orexin system function also presents both challenges and opportunities for intervention. Clinical evidence shows elevated CSF levels of orexin-A in patients with anxiety, while reduced levels are observed in obese individuals [8,13]. This seemingly contradictory pattern actually reflects differential dysregulation of the orexin system under distinct pathological conditions. Accordingly, we hypothesize that treatment of anxiety–obesity comorbidity may require subtype-specific therapeutic strategies. Specifically, comorbidity originating from anxiety-driven obesity with high endogenous orexin levels may benefit from OX1R blockade to suppress OX1R-dependent anxiety and binge eating while sparing OX2R-linked metabolic functions. By contrast, cases featuring low orexin levels, in which obesity precedes comorbid anxiety, may be treated with OX2R agonists to boost arousal and energy expenditure and ameliorate mood without triggering excessive OX1R-mediated food intake [15].

Preclinical evidence has preliminarily validated the druggability of targeting the orexin system. With the dual orexin receptor antagonist suvorexant successfully approved for treating insomnia, laying the foundation for developing additional orexin receptor antagonists and agonists to address other conditions such as anxiety and obesity [105]. Nevertheless, animal studies and preclinical data show inconsistent therapeutic outcomes of orexin-targeted interventions against obesity and anxiety. Regarding weight management, the OX2R agonist YNT-185 significantly activates brown adipose tissue thermogenesis and improves metabolic disturbances in high-fat diet-induced obese mice, whereas another selective OX2R agonist, TAK-925, improved wakefulness in obesity models without demonstrating significant weight reduction [106,107]. In contrast, in anxiety treatment, the dual-receptor antagonist DORA-12 exhibited marked anxiolytic effects in acute stress rat models, while the selective OX1R antagonist SB-334867 only partially alleviated anxiety-like behaviors at high doses in chronic social defeat stress and failed to affect associated social avoidance [8]. These discrepancies may relate to the bidirectional regulation of the orexin system under prolonged metabolic stress and chronic stress: blocking the system is beneficial for anxiety in individuals with elevated baseline orexin levels, but suppressing it in those with lower levels may induce somnolence and reduced motivation, thereby hindering weight loss and emotional improvement.

## 7. Conclusions

Based on current evidence, this review proposes an integrative perspective in which sustained enhancement or circadian dysregulation of endogenous orexin signaling may simultaneously drive metabolic imbalance, increased emotional vulnerability, and altered autonomic regulation, thereby contributing to the development of obesity–anxiety comorbidity. In this context, the orexin system should be viewed not only as a metabolic regulator but also as a central integrative node linking reward processing, stress responses, and emotional regulation.

Future studies should further elucidate the structural specificity and functional connectivity of orexin-related neural circuits, particularly interactions among the LH–VTA–NAcc reward pathway, the BNST–CRH stress circuit, and the SCN-driven circadian regulatory network. Understanding how these circuits dynamically change across physiological states and disease stages will facilitate the identification of novel therapeutic targets and support the development of more precise intervention strategies for obesity and its psychiatric comorbidities. Moreover, directly including male and female individuals in preclinical studies to clarify the impact of the orexin-mediated sex dimorphism on comorbid mechanisms, and conducting cross-species comparative analyses of neural circuits to bridge promising findings from animal models with complex human clinical phenotypes, may promote the clinical translation of orexin-based therapeutic approaches for metabolic–psychiatric comorbid disorders.

## Data Availability

No new data were created or analyzed in this study.
